# Young or Old Age and Non-White Race Are Associated With Poor Patient-Reported Outcome Measure Response Compliance After Orthopaedic Surgery

**DOI:** 10.1016/j.asmr.2023.100817

**Published:** 2023-11-11

**Authors:** Benjamin Levens, Brian Sangwook Kim, Nicholas Aksu, C. Scott Dorris, Steven Svoboda, Wiemi Douoguih, James Dreese

**Affiliations:** a Medstar Union Memorial Hospital, Baltimore, Maryland, U.S.A.; b Georgetown University, Washington, DC, U.S.A.; c Medstar Health at Lafayette Centre, Washington, DC, U.S.A.

## Abstract

**Purpose:**

To investigate orthopaedic patient compliance with patient-reported outcome measures (PROMs) and identify factors that improve response rates.

**Methods:**

Our search strategy comprised a combination of key words and database-specific subject headings for the concepts of orthopaedic surgical procedures, compliance, and PROMs from several research databases from inception to October 11, 2022. Duplicates were removed. A total of 97 studies were included. A table was created for the remaining articles to be appraised and analyzed. The collected data included study characteristics, follow-up/compliance rate, factors that increase/decrease compliance, and type of PROM. Follow-up/compliance rate was determined to be any reported response rate. The range and average used for analysis was based on the highest or lowest number reported in the specific article.

**Results:**

The range of compliance reported was 11.3% to 100%. The overall response rate was 68.6%. The average baseline (preoperative/previsit) response rate was 76.6%. Most studies (77%) had greater than 50% compliance. Intervention/reminder of any type (most commonly phone call or mail) resulted in improved compliance from 44.6% to 70.6%. Young and elderly non-White male patients had the lowest compliance rate. When directly compared, phone call (71.5%) resulted in a greater compliance rate than electronic-based (53.2%) or paper-based (57.6%) surveys.

**Conclusions:**

The response rates for PROMs vary across the orthopaedic literature. Patient-specific factors, such as age (young or old) and race (non-White), may contribute to poor PROM response rate. Reminders and interventions significantly improve PROM response rates.

**Clinical Relevance:**

PROMs are important tools in many aspects of medicine. The data generated from these tools not only provide information about individual patient outcomes but also make hypothesis-driven comparisons possible. Understanding the factors that affect patient compliance with PROMs is vital to our accurate understanding of patient outcomes and the overall advancement of medical care.

Patient-reported outcome measures (PROMs) provide an invaluable resource to the field of medicine. The assessment of outcome from a patient perspective accompanied by that of the clinician creates a more realistic evaluation of quality of care. PROMs standardize subjective responses into an objective measurement, enabling hypothesis-driven comparison. Without PROMs, subjective data are highly heterogeneous, making comparison difficult.^1^

Several studies suggest incorporation of PROMs can improve patient–physician communication and patient outcomes.^1^^,^^2^ As U.S. health care costs increase, many services are under increased cost-cutting scrutiny. This has led to a rapid shift in reimbursement model from traditional volume-driven fee-for-service to value-based payment models.^3^ At the core of this shift is value analysis through PROMs.^4^^,^^5^ PROMs provide another measure to determine cost-effectiveness in health care. For this reason, clinical use of PROMs continues to increase at a rapid pace.^6^

As an objective measurement tool, it is essential for PROMs to have adequate responsiveness, validity, and reliability. On a population level, these qualities have the potential to be significantly affected by patient compliance, as inadequate response rate introduces selection bias and reduces external validity.^1^^,^^3^^,^^6^, ^7^, ^8^ Real-world compliance is multifactorial. Theoretically, variables including specific PROM used, method of admission, clinic staffing, and more may have significant effects on individual study compliance.^1^^,^^9^ Optimization of these variables is a common struggle experienced when incorporating PROMs into practice with no consensus on most important factors to consider.^5^ Due to this inconsistency, general compliance with PROMs in the field of orthopaedics is unknown. There is a paucity of information in the literature evaluating overall compliance regarding PROMs in the field of orthopaedics. Knowing PROM compliance rates is valuable in understanding potential for sampling bias, important factors of consideration in future clinical implementation, policy change, and study design. The purposes of this systematic review are to investigate orthopaedic patient compliance with PROMs and identify factors that improve response rates. Our hypothesis was that compliance to PROM would be poor but could be improved with the use of certain interventions.

## Methods

This systematic review was conducted and reported according to the Preferred Reporting Items for Systematic Reviews and Meta-Analyses guidelines.^10^

### Information Sources and Search Strategy

Our search included MEDLINE, Embase, Cochrane Central Register of Controlled Trials (all via Ovid), Web of Science Core Collection, and SPORTDiscus via EBSCOhost from each database’s inception until October 11, 2022. The search strategy comprised a combination of key words and database-specific subject headings for the concepts of orthopaedic surgical procedures, compliance, and PROMs. In order to capture the largest possible queue of articles, the only exclusion was non-English studies to avoid issues involving English translation. Some examples of key words include a combination of compliance or variations of the word (compliant, comply, complies, etc), PROM (PRO, PROM), specific PROMs (Patient-Reported Outcomes Measurement Information System, 12-Item Short Form Health Survey, Knee injury and Osteoarthritis Outcome Score, Western Ontario and McMaster Universities Arthritis Index, etc.) and orthopaedic surgery (ortho, orthopedic, arthroscopy, arthroplasty).

### Selection and Data-Collection Process

After completion of the query, duplicates were removed using EndNote X9 (Clarivate Analytics, Philadelphia, PA). The identified articles were uploaded to Rayyan (Doha, Qatar) for screening. Initial screening of titles and abstracts for relevance was conducted by 2 independent reviewers (B.S.K., N.E.A.). Each reviewer was blinded to the results of the other to prevent any selection bias. Any discrepancies during the screening or extraction process were resolved by consensus agreement between the reviewers (B.S.K., N.E.A.) and the primary author (B.J.L.). Two separate rounds of review processes were performed. The first review was broader, including any study pertaining to orthopaedic surgery and PROMs. The second review was narrower, including only articles that specifically mentioned PROM compliance. Full texts of the remaining articles were obtained and assessed for eligibility by the same 2 independent reviewers in addition to the primary author.

### Data Items

The information gathered from the systematic review was compiled into a table. The information included study characteristics, follow-up/compliance rate, factors that increase/decrease compliance, and type of PROM. Follow-up/compliance rate was determined to be any reported response rate. If different modalities were used in the study, those were included in the table. The range and average used for analysis was based on the highest or lowest number reported in the specific article.

## Results

### Study Selection

**Fig 1:**
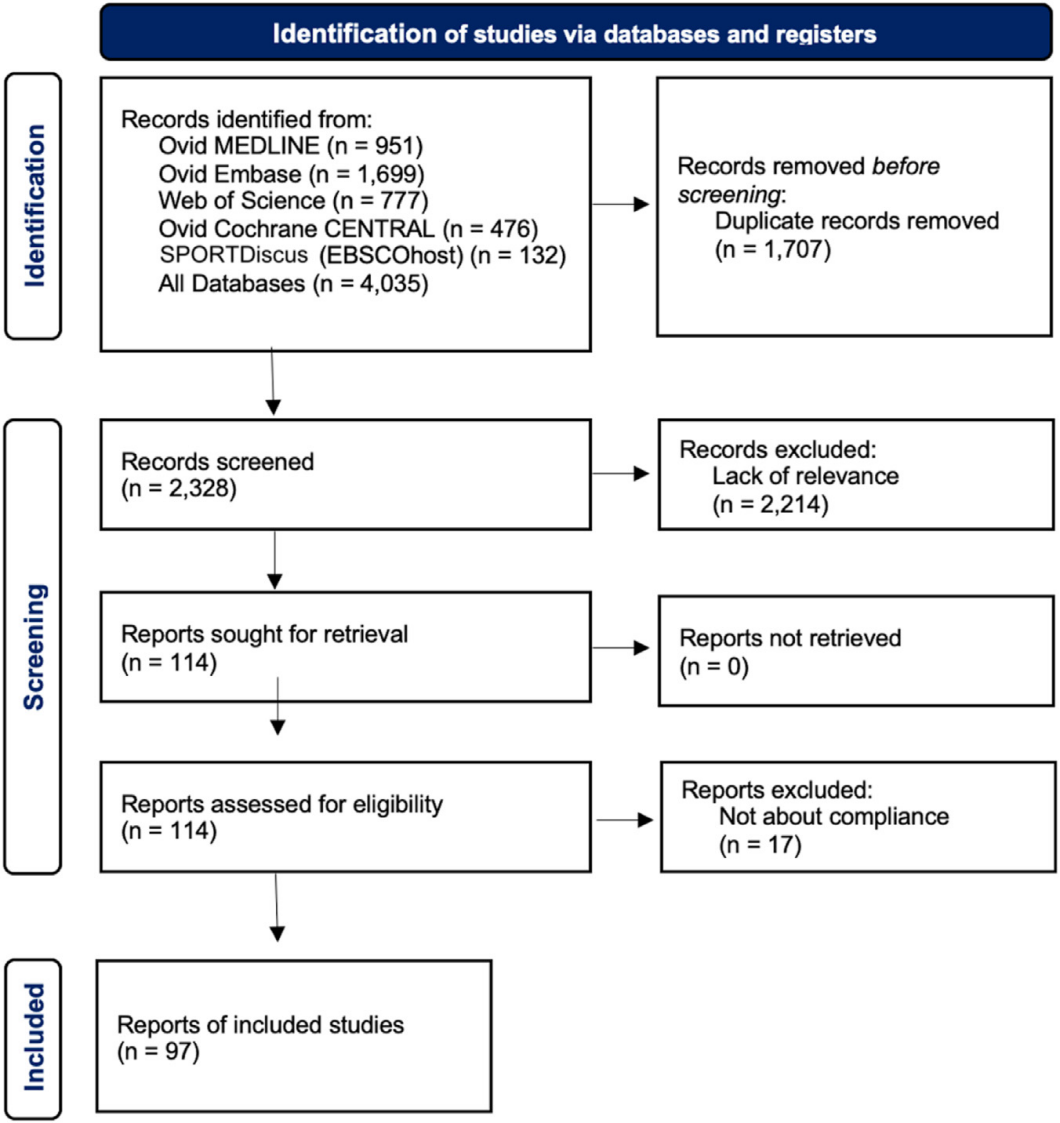
Flow diagram of search query.

The initial search yielded 4,035 citations. After removal of duplicates, 2,328 citations remained. After the first, broader screening, 1,500 citations remained. On the second, narrower screening, 97 were included (Table 1).^11^, ^12^, ^13^, ^14^, ^15^, ^16^, ^17^, ^18^, ^19^, ^20^, ^21^, ^22^, ^23^, ^24^, ^25^, ^26^, ^27^, ^28^, ^29^, ^30^, ^31^, ^32^, ^33^, ^34^, ^35^, ^36^, ^37^, ^38^, ^39^, ^40^, ^41^, ^42^, ^43^, ^44^, ^45^, ^46^, ^47^, ^48^, ^49^, ^50^, ^51^, ^52^, ^53^, ^54^, ^55^, ^56^, ^57^, ^58^, ^59^, ^60^, ^61^, ^62^, ^63^, ^64^, ^65^, ^66^, ^67^, ^68^, ^69^, ^70^, ^71^, ^72^, ^73^, ^74^, ^75^, ^76^, ^77^, ^78^, ^79^, ^80^, ^81^, ^82^, ^83^, ^84^, ^85^, ^86^, ^87^, ^88^, ^89^, ^90^, ^91^, ^92^, ^93^, ^94^, ^95^, ^96^, ^97^, ^98^, ^99^, ^100^, ^101^, ^102^, ^103^, ^104^, ^105^, ^106^, ^107^ A flow diagram of the screening process is included in Figure 1.

**Table 1:** Literature Review of the 97 Included Citations

Title	First Author	Journal	Year	PubMed ID (if Applicable)	Type of Study	Preoperative	Highest Reported/Postoperative	Patient Factors That Increase Compliance	Patient Factors That Decrease Compliance	Provider Intervention
Relationship of Press Ganey Satisfaction and PROMIS Function and Pain in Foot and Ankle Patients	Nixon^40^	*Foot Ankle Int*	2020	32660263	Retrospective chart review		11.3			
Response Bias for Press Ganey Ambulatory Surgery Surveys after Knee Surgery	Zhang^41^	*J Knee Surg*	2022	35817060	Prospective cohort		12.2		Male, non-White, student or unemployment status, and worse preoperative score	
Press Ganey Surveys in Patients Undergoing Upper-Extremity Surgical Procedures: Response Rate and Evidence of Nonresponse Bias^35^	Weir^35^	*Journal of Bone & Joint Surgery*	2021	33988529	Retrospective chart review		13.5	White, higher education, current employment, and married		
Two Years Following Implementation of the British Spinal Registry (BSR) in a District General Hospital (DGH): Perils, Problems and PROMS	Roysam^42^	*Spine Journal*	2016		Prospective cohort	62	20			
Evaluation of the Implementation of PROMIS CAT Batteries for Total Joint Arthroplasty in an Electronic Health Record	Rothrock^43^	*Quality of Life Research*	2018		Prospective cohor study		31.8			
Factors Associated With Survey Response in Hand Surgery Research^16^	Bot^16^	*Clinical Orthopedics and Related Research*	2013	23801062	Prospective cohort study		34		Male, younger age, higher pain, and worse preoperative score	
Two and a Half Years On: Data and Experiences Establishing a 'Virtual Clinic' for Joint Replacement Follow Up	Lovelock^44^	*ANZ Journal of Surgery*	2018	29952097	Prospective cohort		35			
Association Between Patient Factors and Hospital Completeness of a Patient-Reported Outcome Measures Program in Joint Arthroplasty, A Cohort Study	Harris^45^	*Journal of Patient-Reported Outcomes*	2022	35380301	Multicenter cohort study		36.3			
Comparison of Paper and Electronic Surveys for Measuring Patient-Reported Outcomes After Anterior Cruciate Ligament Reconstruction	Bojcic^46^	*Permanente Journal*	2014	25102515	Cross-sectional study		36.3			
Level of Response to Telematic Questionnaires on Health Related Quality of Life on Total Knee Replacement	Besalduch-Balaguer, M^47^	*Revista Espanola de Cirugia Ortopedica y Traumatologia*	2015	25435294	Observational	37				
Differences in Baseline Characteristics and Outcome Among Responders, Late Responders, and Never-Responders After Anterior Cruciate Ligament Reconstruction^19^	Randsborg, PH^19^	*The American Journal of Sports Medicine*	2021	34723674	Case–control study		40		Younger age, male, low education (high school or less), and non-White	
Sociodemographic Factors Are Associated With Patient-Reported Outcome Measure Completion in Orthopaedic Surgery: An Analysis of Completion Rates and Determinants Among New Patients^32^	Bernstein DN^32^	*JB & JS Open Access*	2022	35935603	Retrospective observational study	40		Older age (>65 y), non-White, and non-English speaking		
Collection and Reporting of Patient-Reported Outcome Measures in Arthroplasty Registries: Multinational Survey and Recommendations	Bohm, ER^48^	*Clinical Orthopedics and Related Research*	2021	34288899	Cross-sectional descriptive study	40				
Male Sex, Decreased Activity Level, and Higher BMI Associated With Lower Completion of Patient-Reported Outcome Measures Following ACL Reconstruction^38^	Cotter^38^	*Orthopaedic Journal of Sports Medicine*	2018	29536023	Prospective survey	7.4	40.6	Lower BMI		
E-mail Reminders Improve Completion Rates of Patient-Reported Outcome Measures	Triplet JJ^49^	*Journal of Shoulder & Elbow Surgery*	2017	30675535	Retrospective cohort study		40.9			Email reminders improved response rate
Pre-visit Digital Messaging Improves Patient Reported Outcome Measure Participation Prior to the Orthopedic Ambulatory Visit^13^	Yedulla^13^	*J Bone Joint Surg Am*	2022	36598473	Prospective RCT		44			Previsit e-mail or patient portal messages resulted in greater completion rate
Small Social Incentives Did Not Improve the Survey Response Rate of Patients Who Underwent Orthopaedic Surgery: A Randomized Trial^11^	Warwick^11^	*Clin Orthop Relat Res*	2019	31135552	Prospective randomized controlled trial	46	Female, older age, and White			
Do Medicare’s Patient-Reported Outcome Measures Collection Windows Accurately Reflect Academic Clinical Practice?	Molloy IB^50^	*The Journal of Arthroplasty*	2020	31889578	Retrospective cohort analysis	46.2				
What Factors Are Associated With Patient-reported Outcome Measure Questionnaire Completion for an Electronic Shoulder Arthroplasty Registry?	Ling DI^51^	*Clinical Orthopaedics & Related Research*	2021	32740479	Retrospective cohort	72	47			Phone call or e-mail reminder from a research assistant
Factors Associated With Early Postoperative Survey Completion in Orthopaedic Surgery Patients^34^	Sajak PM^34^	*Journal of Clinical Orthopedics and Trauma*	2020	31992938	Retrospective cohort study		48	Never smokers, higher education (college), White, married, employment, higher income, private insurance		
Remote Collection of Patient-Reported Outcomes Following Outpatient Hand Surgery: A Randomized Trial of Telephone, Mail, and E-Mail^15^	Schwartzenberger^15^	*J Hand Surg Am*	2017	28600107	Prospective randomized trial		48	Older age and private insurance		
What Factors Are Associated With Response Rates for Long-term Follow-up Questionnaire Studies in Hand Surgery	Westenberg RF^52^	*Clinical Orthopaedics & Related Research*	2020	32452929	Prospective cohort		49			Phone call to nonresponders
The Effects of a Pandemic on Patient Engagement in a Patient-Reported Outcome Platform at Orthopaedic Sports Medicine Centers	Barnds B^53^	*Orthopaedic Journal of Sports Medicine*	2021	PMC8562621	Retrospective cohort study		50.95			
A Non-Response Analysis of 2-YEAR DATA in the Swedish Knee Ligament Register^20^	Reinholdsson, J^20^	*Knee Surgery, Sports Traumatology, Arthroscopy*	2016	26724828	Retrospective cohort analysis	52	Older age and female			
Utilization of an Automated SMS-Based Electronic Patient-Reported Outcome Tool in Spinal Surgery Patients	Elsabeh R^54^	The Spine Journal (34th NASS meeting)	2021		Retrospective cohort		52			
Barriers to Completion of Patient Reported Outcome Measures^28^	Schamber EM^28^	*The Journal of Arthroplasty*	2013	23890831	Prospective cohort study		54.5		Older age (>75 y), non-White, revision surgery, non-private insurance (Medicare and Medicaid)	
Implementation of an Automated Text Message-Based System for Tracking Patient-Reported Outcomes in Spine Surgery: An Overview of the Concept and Our Early Experience	Perdomo-Pantoja, A^55^	*World Neurosurgery*	2022	34800733	Prospective cohort	71.2	54.9			
Management of Distal Radius Fractures in the Emergency Department: A Long-Term Functional Outcome Measure Study With the Disabilities of Arm, Shoulder and Hand (DASH) Scores	Barai, A^56^	*EMA - Emergency Medicine Australasia*	2018	29488343	Prospective cohort		56			
Patient Demographic and Surgical Factors That Affect Completion of Patient-Reported Outcomes 90 Days and 1 Year After Spine Surgery: Analysis From the Michigan Spine Surgery Improvement Collaborative (MSSIC)^21^	Zakaria H^21^	*World Neurosurgery*	2019	31207366	Prospective cohort	72.6	56.3	Older age, higher education, and female		
Patient Compliance With Electronic Patient Reported Outcomes Following Shoulder Arthroscopy	Makhni E^57^	*Arthroscopy*	2017	28958797	Prospective cohort	76	57			Research assistant
Continued Good Results With Modular Trabecular Metal Augments for Acetabular Defects in Hip Arthroplasty at 7 to 11 Years	Whitehouse MR^58^	*Clinical Orthopedics and Related Research*	2015	25123241	Retrospective cohort study		58			
The Danish Hip Arthroscopy Registry: Registration Completeness and Patient Characteristics Between Responders and Non-Responders^22^	Poulsen E^22^	*Clinical Epidemiology*	2020	32801920	Retrospective cohort study		58		Younger age (<25 y) and male	
Overview of the AOA National Joint Replacement Registry: ACL Registry Pilot Study	Clarnette R^59^	*Orthopaedic Journal of Sports Medicine*	2015	PMC4901772	Pilot prospective cohort		58.5			
Evaluating the Measures in Patient-Reported Outcomes, Values and Experiences (EMPROVE study): A Collaborative Audit of PROMs Practice in Orthopaedic Care in the United Kingdom	Matthew A^60^	*The Annals of The Royal College of Surgeons of England*	2022	35938506	Multicenter retrospective cohort study	60				
Collection of Common Knee Patient-reported Outcome Instruments by Automated Mobile Phone Text Messaging in Pediatric Sports Medicine^18^	Mellor X^18^	*Journal of Pediatric Orthopedics*	2020	31107346	Prospective cohort study		60.4	Female, older age, younger age (<18 y)		
An Exploratory Study of Response Shift In Health-Related Quality of Life and Utility Assessment Among Patients With Osteoarthritis Undergoing Total Knee Replacement Surgery in a Tertiary Hospital in Singapore^24^	Zhang XH^24^	*Value in Health*	2012	22265071	Prospective cohort study		63			
A Last-Ditch Effort and Personalized Surgeon Letter Improves PROMs Follow-Up Rate in Sports Medicine Patients: A Crossover Randomized Controlled Trial^12^	Tariq MB	*The Journal of Knee Surgery*	2019	31390674	Crossover RCT		65			Personalized surgeon letter
Automated Reporting of Patient Outcomes in Hand Surgery: A Pilot Study	Franko OI	*Hand*	2022	34521230	Prospective cohort study		65			
The Patient Perspective on Patient-Reported Outcome Measures Following Elective Hand Surgery: A Convergent Mixed-Methods Analysis	Shapiro LM	*Journal of Hand Surgery*	2021	33183858	Prospective cohort study		66			
The Remote Completion Rate of Electronic Patient-Reported Outcome Forms Before Scheduled Clinic Visits-A Proof-of-Concept Study Using Patient-Reported Outcome Measurement Information System Computer Adaptive Test Questionnaires^23^	Borowsky PA	*Journal of the American Academy of Orthopaedic Surgeons Global Research and Reviews*	2019	31773074	Prospective cohort study		67	Female, White, higher income		
Multidisciplinary Rehabilitation or Surgery for Chronic Low Back Pain—7 Year Follow Up of a Randomised Controlled Trial	Barker K	*Spine*	2010		Prospective cohort		67			
Evaluating Non-responders of a Survey in the Swedish Fracture Register: No Indication of Different Functional Result^17^	Juto H	*BMC Musculoskeletal Disorders*	2017	28659134	Prospective cohort study		68	Women, older age (>60 y)		Phone call
Integration of Patient-reported Outcomes in a Total Joint Arthroplasty Program at a High-volume Academic Medical Center	Bhatt	*JAAOS: Global Research and Reviews*	2020	33970573	Prospective cohort		68			
Feasibility of Web-Based Patient-Reported Outcome Assessment After Arthroscopic Knee Surgery: The Patients' Perspective	Olach M	*Swiss Medical Weekly*	2021		Prospective cohort		69.6			
Interpretations of the Clinical Outcomes of the Nonresponders to Mail Surveys in Patients After Total Knee Arthroplasty	Kwan	*Journal of Arthroplasty*	2010	19106032	Prospective cohort		69.8		Worse preoperative score	
The RaCeR Study: Rehabilitation Following Rotator Cuff Repair^14^	Littlewood C	*Clinical Rehabilitation*	2021	33305619	Multicenter RCT		71			
Patient-Reported Outcomes After Total Hip and Knee Arthroplasty: Comparison of Midterm Results	Wylde V	*Journal of Arthroplasty*	2009	18534427	Cross sectional survey		72			
Implementing an Electronic Patient-Based Orthopaedic Outcomes System: Factors Affecting Patient Participation Compliance	Tokish	*Military Medicine*	2017	28051984	Prospective cohort		73			Staff intervention
Preoperative Factors Associated with 2-Year Postoperative Survey Completion in Knee Surgery Patients^36^	Kadiyala	*J Knee Surg*	2022	33545724	Prospective cohort	73	73		Smoker and Non-White	
Standard of Care PRO Collection Across a Healthcare System	Rubery P	25th Annual Conference of the International Society for Quality of Life Research	2018		Retrospective study		74			
Age Significantly Affects Response Rate to Outcomes Questionnaires Using Mobile Messaging Software^26^	Jildeh TR	*Arthroscopy, Sports Medicine, and Rehabilitation*	2021	34712973	Prospective cohort study		75		Older age	
Partial Versus Total Trapeziectomy Thumb Arthroplasty: An Expertise-Based Feasibility Study	Thoma A	*Plastic and Reconstructive Surgery - Global Open*	2018	29707461	Prospective cohort		75			
Follow-up Compliance and Outcomes of Knee Ligamentous Reconstruction or Repair Patients Enrolled in an Electronic Versus a Traditional Follow-up Protocol	Shu H	*Orthopedics*	2018	30168836	Retrospective chart review		76			
Active Living With Osteoarthritis Implementation of Evidence-Based Guidelines as First-Line Treatment for Patients With Knee and Hip Osteoarthritis	Risberg M	*Osteoarthritis and Cartilage*	2018		Prospective cohort study		77			
A Pilot Study Investigating the use of At-Home, Web-Based Questionnaires Compiling Patient-Reported Outcome Measures Following Total Hip and Knee Replacement Surgeries	Gakhar H	*Journal of Long-term Effects of Medical implants*	2013	24266443	Prospective cohort study		78			
Polytrauma and High-energy Injury Mechanisms are Associated With Worse Patient-reported Outcomes After Distal Radius Fractures	van der Vliet, Q	*Clinical Orthopaedics & Related Research*	2019	30985610	Retrospective chart review with follow up survey	78				
Feasibility of Collecting Multiple Patient-Reported Outcome Measures Alongside the Dutch Arthroplasty Register	Tilbury C	*Journal of Patient Experience*	2020	33062868	Prospective observational cohort study	78.5				
Patient-Reported Outcome After Displaced Femoral Neck Fracture: A National Survey of 4467 Patients	Leonardsson O	*Journal of Bone & Joint Surgery*	2013	24048557	Prospective cohort		79			Reminder
Combined Email and in Office Technology Improves Patient Reported Outcomes Collection in Standard Orthopaedic Care^33^	Zhou X	*Osteoarthritis and Cartilage*	2014		Prospective cohort study		79	Older Age		
Feasibility of Four Patient Reported Outcome Measures in the Danish Hip Arthroplasty Registry. A Cross-Sectional Study of 6000 Patients	Paulsen	*HIP International*	2010	26625504	Cross-sectional cohort		80			Two reminders sent to nonresponders
Improving the Response Rate of Patient-Reported Outcome Measures in an Australian Tertiary Metropolitan Hospital	Ho	*Patient Related Outcome Measures*	2019	31372076	Prospective cohort		81.01			Paper forms, multi-lingual, staff assistance
Implementing an ICHOM Standard Set to Capture Osteoarthritis Outcomes in Real-World Clinical Settings	Cavka	*Osteoarthritis and Cartilage*	2018	30148249	Mixed-methods design	61	81.6			
Reliability of Patient-Reported Functional Outcome in a Joint Replacement Registry. A Comparison of Primary Responders and Non-responders in the Danish Shoulder Arthroplasty Registry^39^	Polk	*Acta Orthop*	2013	23343374	Prospective cohort		82			Postal reminders
Response Rate and Costs for Automated Patient-Reported Outcomes Collection Alone Compared to Combined Automated and Manual collection	Pronk	*J Patient Rep Outcomes*	2019	31155689	Observational	100	83			Postal reminders
Feasibility of 4 Patient-Reported Outcome Measures in a Registry Setting^27^	Paulsen A	*Acta Ortopaedica*	2012	22900909	Cross-sectional study		84		Older age	
Detailing Postoperative Pain and Opioid Utilization After Periacetabular Osteotomy With Automated Mobile Messaging	Hajewski C	*Journal of Hip Preservation Surgery*	2019	33354334	Single-center prospective cohort study	84.1			Mobile messaging	
Loss to Patient-Reported Outcome Measure Follow-Up After Hip Arthroplasty and Knee Arthroplasty: Patient Satisfaction, Associations With Non-Response, and Maximizing Returns	Ross	*Bone & Joint Open*	2022	35357243	Prospective cohort		84.2			
External Validation of the Tyrolean Hip Arthroplasty Registry^31^	Wagner M	*Journal of Experimental Orthopaedics*	2022	36042064	Cohort	84.45	Younger and male			
Informed, Patient-Centered Decisions Associated With Better Health Outcomes in Orthopedics: Prospective Cohort Study	Sepucha	*Medical Decision Making*	2018	30403575	Observational survey	70.3	85			Phone and mailed reminders
Symptoms of Post-Traumatic Osteoarthritis Remain Stable up to 10 Years After ACL Reconstruction	Spindler K	*Orthopaedic Journal of Sports Medicine*	2022	PMC9339818	Multicenter retrospective cohort study	85				
The Value of Short and Simple Measures to Assess Outcomes for Patients of Total Hip Replacement Surgery	Fitzpatrick R	*Quality in Health Care*	2000	10980074	Retrospective cohort		85.2			
Arthroplasty Studies With Greater Than 1000 Participants: Analysis of Follow-Up Methods	Tariq MB^12^	*Arthroplasty Today*	2019	31286051	Systematic review & meta-analysis	86				
Patient Adoption and Utilization of a Web-Based and Mobile-Based Portal for Collecting Outcomes After Elective Orthopedic Surgery	Bell, K^86^	*American Journal of Medical Quality*	2018	29562769	Retrospective chart review		87.14			
Is It Too Early to Move to Full Electronic PROM Data Collection? A Randomized Controlled Trial Comparing PROM's After Hallux Valgus Captured by E-Mail, Traditional Mail and Telephone	Palmen^87^	Foot and Ankle Surgery	2016	26869500	Prospective cohort		88			
Integrating PROM Collection for Shoulder Surgical Patients through the Electronic Medical Record: A Low Cost and Effective Strategy for High Fidelity PROM Collection	Fife^88^	*Orthopaedic Journal of Sports Medicine*	2022	PMC9339844	Retrospective chart review		88			
Extending the Use of PROM Scores in the Hip and Knee Replacemnt Patient Pathway in the NHS–Enhancing Response Rates Through Patient Engagement	Harris K^89^	Health and Quality of Life Outcomes. Conference: Patient Reported Outcome Measure's, PROMs Conference: Advances in Patient Reported Outcomes Research.	2017	23965934	Prospective cohort study		90			
Implementation of Patient-Reported Outcomes Measurement Information System Data Collection in a Private Orthopedic Surgery Practice	Haskell^90^	*Foot & Ankle International*	2018	29366343	Retrospective chart review		90			
The Oxford Knee Score; Problems and Pitfalls	Whitehouse SL^91^	*The Knee*	2005	15993604	Retrospective cohort study		90			
Factors Affecting the Quality of Life After Total Knee Arthroplasties: A Prospective Study	Papakostidou, I^92^	*BMC Musculoskeletal Disorders*	2012	22748117	Prospective cohort study		90.12			
MOON's Strategy for Obtaining Over Eighty Percent Follow-up at 10 Years Following ACL Reconstruction	Marx R^93^	*Journal of Bone & Joint Surgery*	2022	34424872	Prospective cohort		90.5			Email and telephone calls
Feasibility of PROMIS CAT Administration in the Ambulatory Sports Medicine Clinic With Respect to Cost and Patient Compliance: A Single-Surgeon Experience^29^	Lizzio VA^29^	*Orthopaedic Journal of Sports Medicine*	2019	30733973	Prospective cohort		91.3		Older age	
Cervical Disc Arthroplasty for Degenerative Disc Disease: Two-Year Follow-Up from an International Prospective, Multicenter, Observational Study	Baeesa, SS^94^	*The Spine Journal*	2015		Observational	92				
Internet-Based Follow-Up Questionnaire for Measuring Patient-Reported Outcome After Total Hip Replacement Surgery-Reliability and Response Rate	Rolfson^95^	*Value in Health*	2011	21402299	Prospective cohort		92			
PROMIS Physical Function Correlation With NDI and mJOA in the Surgical Cervical Myelopathy Patient Population	Owen^96^	*Spine (Phila Pa 1976)*	2018	28787313	Prospective cohort	100	92			
What Is the Minimum Response Rate on Patient-Reported Outcome Measures Needed to Adequately Evaluate Total Hip Arthroplasties	Pronk Y^97^	*Health and Quality of Life Outcomes*	2020	33267842	Retrospective cohort	99.8	92.2			Phone call
Mobile Phone Administration of Hip-Specific Patient-Reported Outcome Instruments Correlates Highly With In-office Administration	Scott E^98^	*Journal of the American Academy of Orthopaedic Surgeons*	2020	31860543	Prospective cohort		93			Text message
Validation of Electronic Administration of Knee Surveys Among ACL-Injured Patients	Nguyen J^99^	*Knee Surgery, Sports Traumatology, Arthroscopy*	2017	27316698	Prospective cohort		94			
PROMIS Correlation With NDI and VAS Measurements of Physical Function and Pain in Surgical Patients With Cervical Disc Herniations and Radiculopathy	Owen^100^	*J Neurosurg Spine*	2019	31277059	Prospective cohort	100	94			
Prospective Randomized Cohort Study to Explore the Acceptability of Patient-Reported Outcome Measures to Patients of Hand Clinics	Sierakowski^101^	*J Hand Surg Glob Online*	2020	35415526	Prospective randomized cohort	85	94			
Perioperative Satisfaction and Health Economic Questionnaires in Patients Undergoing an Elective Hip and Knee Arthroplasty: A Prospective Observational Cohort Study	Nagappa, M^102^	*Anesthesia: Essays and Researches*	2021	35422546	Prospective cohort	98.8	94.2			
Networking to Capture Patient-Reported Outcomes During Routine Orthopaedic Care Across Two Distinct Institutions	Karia R^103^	*Osteoarthritis and Cartilage*	2013		Prospective cohort		95			
Feasibility of Integrating Standardized Patient-Reported Outcomes in Orthopedic Care	Slover J^104^	*American Journal of Managed Care*	2015	26625504	Prospective cohort		95			
Patient Satisfaction Compared With General Health and Disease-Specific Questionnaires in Knee Arthroplasty Patients^30^	Robertsson O^30^	*Journal of Arthroplasty*	2001	11402411	Survey		95.1		Older age, female, and worse preoperative score	
Monitoring Patient Recovery After THA or TKA Using Mobile Technology	Lyman S^105^	*HSS Journal*	2020	33380968	Prospective cohort		96			
The Use of a Patient-Based Questionnaire (The Oxford Shoulder Score) to Assess Outcome After Rotator Cuff Repair	Olley LM^106^	*The Annals of The Royal College of Surgeons of England*	2008	18492399	Prospective cohort		97			Phone call
A Descriptive Study of the Use of Visual Analogue Scales and Verbal Rating Scales for the Assessment of Postoperative Pain in Orthopedic Patients^25^	Briggs M^25^	*Journal of Pain and Symptom Management*	1999	10641470	Prospective cohort study		99.5		Older age and Female	
Short Message Service-Based Collection of Patient-Reported Outcome Measures on Hand Surgery Global Outreach Trips: A Pilot Feasibility Study	Shapiro^107^	J *Hand Surg Am*	2022	34148790	Prospective cohort		100			

ACL, anterior cruciate ligament; PROM; patient-reported outcome measure; RCT, randomized controlled trial; THA, total hip arthroplasty; TKA, total knee arthroplasty.

### Study Characteristics

The 97 included citations were published between 1999 and 2022; 93.8% (91/97) were published after 2010. In total, 94.8% (92/97) of citations were nonrandomized observational studies.

### Overall Compliance

All 97 studies reported PROM response in either the postoperative/postvisit setting or did not specify. The mean response rate overall was 68.6% (range 11.3%-100%). The median response rate was 73%. In total, 77% (75/97) of studies had greater than 50% compliance.

### Baseline (Preoperative or Previsit)

Only 15% (15/97) reported PROM response in the preoperative/previsit setting. The mean response rate across these studies was 76.6% (range 7.4%-100%). The median response rate was 73%. In those 15 studies that included preoperative/previsit baseline PROMs, the mean response rate of PROM in the postoperative/postvisit setting for those particular studies was 71% (range 40.6%-94.2%).

### Results by Study Type

In total, 5.2% (5/97) of publications were randomized controlled trials (RCTs). Of the 5, 4 studies had PROM as the primary outcome measure for randomization.^11^, ^12^, ^13^, ^14^ The 4 studies aimed to identify what factors improved response rate either compared with a control or to different modalities. The mean response rate among the RCTs was 54.8% (range 44%-71%, median 48%).

One RCT directly compared response rate based on different collection methods: phone call, e-mail, or mail.^15^ The overall response rate for the study was 48%. Phone calls yielded the greatest response rate of 64% versus 42% for e-mail and 42% for mail. In total, 94.8% (92/97) of citations were nonrandomized observational studies. The mean response rate among these studies was 69.4% (range 11.3%-100%). The median response rate was 75%.

### Intervention

**Fig 2:**
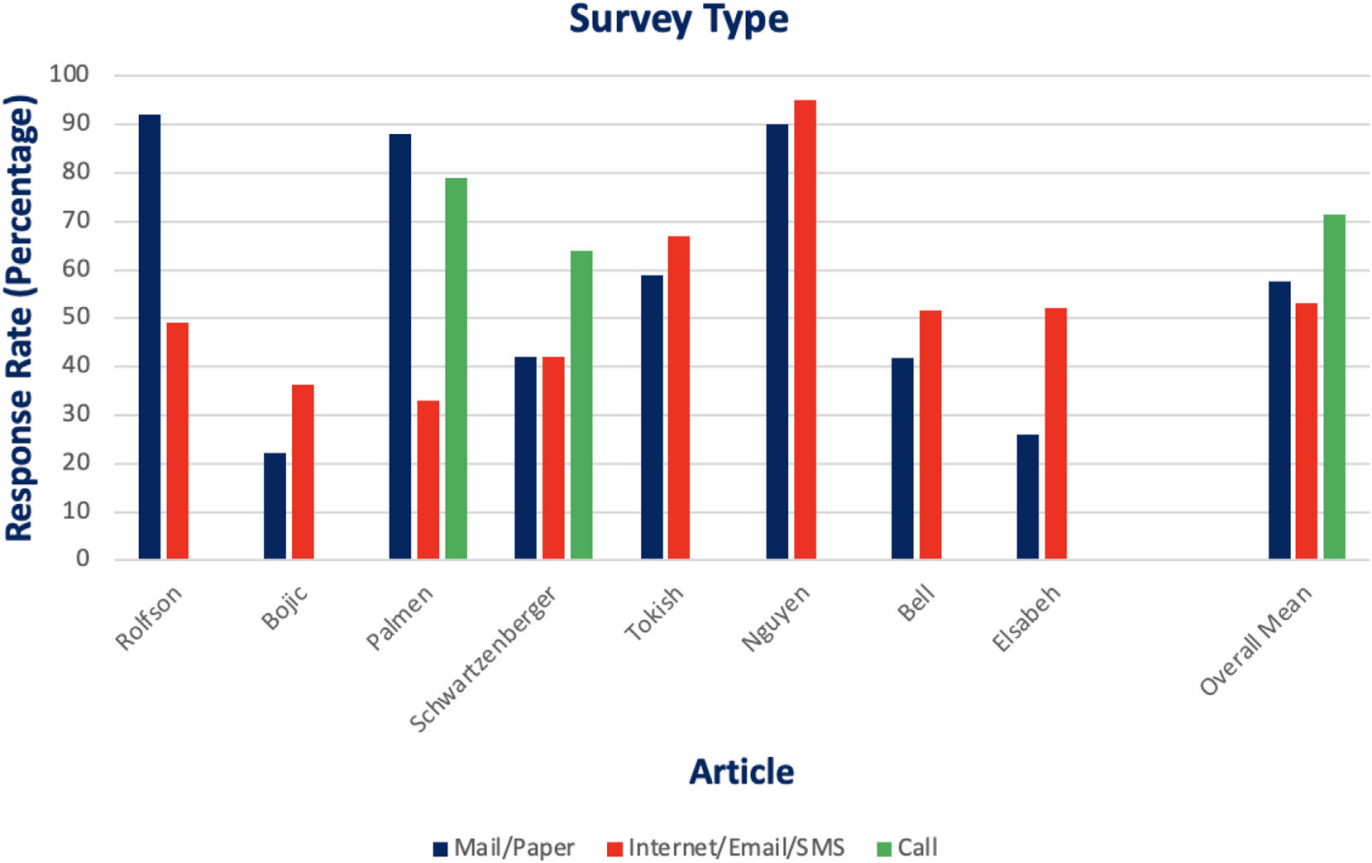
Graph of response rate by survey type from the articles that were directly compared.

Intervention/reminder of any type (most commonly phone call or mail) resulted in improved compliance from 44.6% to 70.6%. Reminder types included phone call, mail, e-mail, text message, or some combination of multiple. When directly compared, phone call (71.5%) resulted in a greater compliance rate than electronic-based (53.2%) or paper-based (57.6%) surveys. The findings are shown in Figure 2.

### Patient-Specific Factors

**Fig 3:**
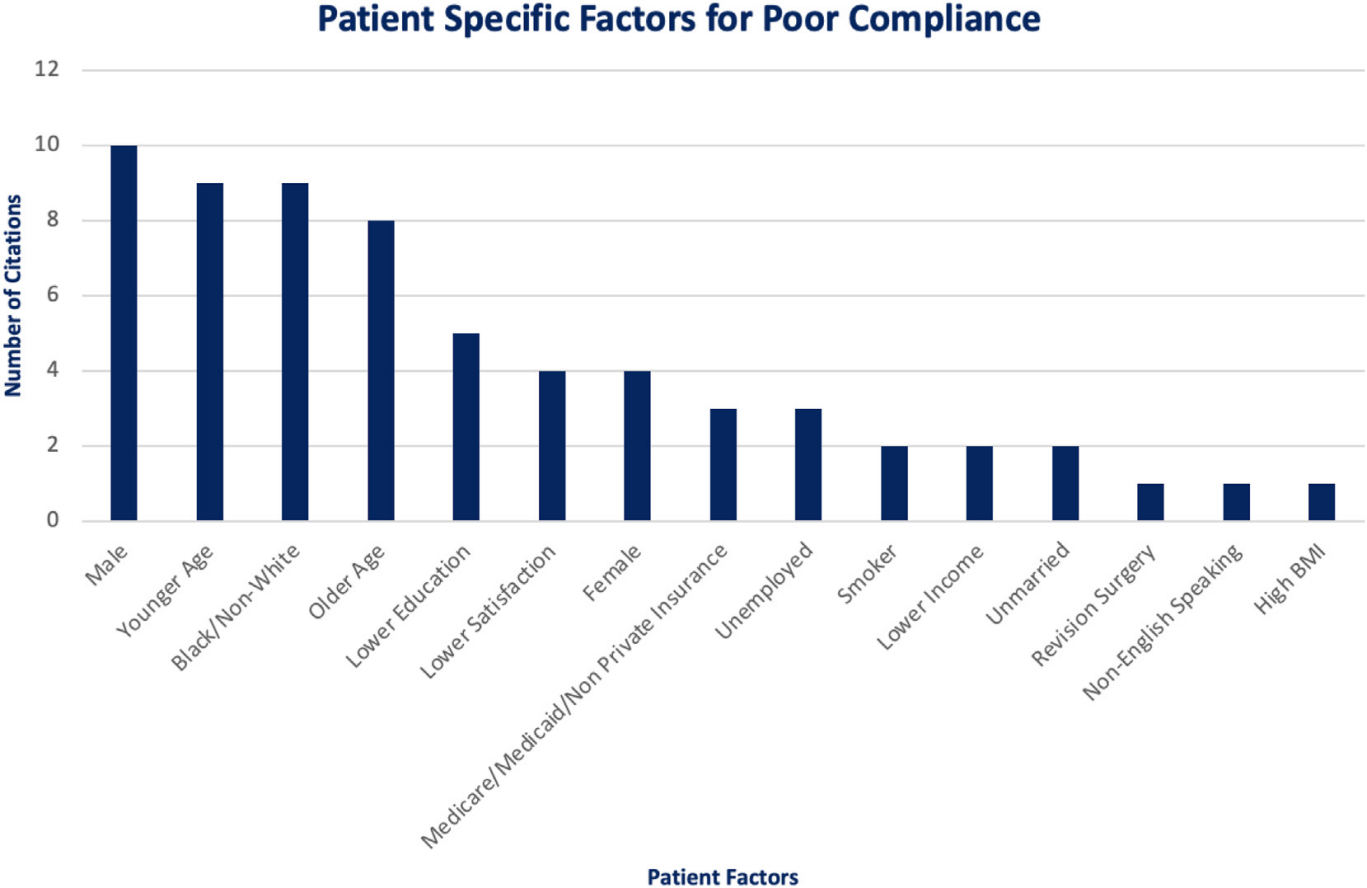
Graph of patient-specific factors cited as contributing to poor compliance. (BMI, body mass index.)

There were many different demographic characteristics compared in individual studies. Age, sex, race, education, insurance type, employment, smoking status, satisfaction rate, marital status, body mass index, and primary language were some of the demographics collected. Although there was heterogeneity in the results, the most commonly contributed factors to poor compliance were male sex,^11^^,^^16^, ^17^, ^18^, ^19^, ^20^, ^21^, ^22^, ^23^, ^24^ extremes of age (young and old),^11^^,^^15^^,^^16^^,^^18^, ^19^, ^20^, ^21^, ^22^^,^^25^, ^26^, ^27^, ^28^, ^29^, ^30^, ^31^, ^32^, ^33^ and non-White race.^11^^,^^19^^,^^24^^,^^28^^,^^32^^,^^34^, ^35^, ^36^ Lower education, lower satisfaction, female sex, nonprivate insurance, unemployed, smoker, lower income, prior surgery, unmarried, high body mass index and non-English-speaking were some of the other factors mentioned in individual citations to be associated with poor compliance.^15^^,^^16^^,^^19^^,^^21^^,^^23^, ^24^, ^25^^,^^28^^,^^30^, ^31^, ^32^, ^33^, ^34^, ^35^, ^36^, ^37^, ^38^ These findings are shown in Figure 3.

## Discussion

The most important finding of this systematic review is that although a variety of factors can affect compliance with PROMS after orthopaedic surgery, reminders and other interventions can improve response rates. All 97 studies included in this systematic review reported PROM response rate in the postoperative setting. The average response rate across these studies was 53.6% (range 11.3%-100%). In addition to PROMs in the postoperative setting, it is crucial to obtain PROMs in the preoperative setting. Doing so establishes a baseline score for objective comparison to determine whether a surgical intervention was successful. Ideally, the rate of compliance in the postoperative setting should be similar or improved as compared with compliance in the preoperative setting.

Of the 97 studies that reported PROM compliance in the postoperative period, only 15% reported PROM response in the preoperative setting. The average response rate across these studies was 76.6% (range 7.4%-100%). When further examining the rate of PROM response in the postoperative setting for these 15 studies, the average response rate was 71% (range 40.6%-94.2%). Overall, the average PROM response rate in the postoperative setting for all included studies was 68.6% (range 11.3%-100%).

The compliance rates in PROMs poses several issues when evaluating the validity of an orthopaedic study. One particular concern is the introduction of response bias when patients are lost to follow-up. This could be attributed to a spectrum of reasons. One reason being these patients may experience worse outcomes in pain and function that discourage them from continued follow-up. In fact, 4 of the evaluated studies cited lower patient satisfaction as one of the reasons for decreased rates of PROM compliance. Socioeconomic and demographic factors may also play a role, as a number of the evaluated studies cited male sex, older age, non-White race, lower education, and lower income or unemployed backgrounds as risk factors for poor compliance. The cumulative effect of these factors introduces significant bias in what is supposed to serve as an objective measurement tool in PROMs. Thus, this highlights the added importance of maintaining high rates of compliance in PROMs in order to preserve an appropriate level of study validity and reliability.

A commonly used method to increase PROM compliance is the use of reminders. In a study by Polk et al.^39^ that observed PROM responsiveness in the Danish Shoulder Arthroplasty Registry, it was reported that the rate of response at the 1-year mark for follow-up was 65% before the use of a reminder. They then used mail-only and call/mail reminders to initial nonresponders, and subsequently observed response rates of 80% and 82% respectively.

PROM compliance also may depend on the mode of communication in which it is presented to patients. PROMs may be obtained with the use of surveys delivered via electronic or non–electronic-based methods. This can include phone calls, mail or paper surveys, e-mail surveys, or SMS (ie, Short Message/Messaging) responses. Overall, an intervention of any type demonstrated improvement in response rate from an average of 44.6% to 70.6% across all studies that used an intervention. Upon further analysis across 8 studies that used phone call-, electronic-, and mail-based interventions, phone call demonstrated the greatest compliance rate (71.5%) as compared with paper (57.6%) or electronic (53.2%). In a study by Schwartzenberger et al.^15^ that implemented an RCT comparing phone, e-mail, and mail, they observed similar results, with telephone PROM collection having the greatest rate of compliance (64%) as compared with e-mail or mail (42% each). This may demonstrate the impact of personalized follow-up on compliance. Patients may feel more inclined to fill out a PROM survey when they are being directly asked.

Another consideration is that PROM surveys often contain medical jargon that is unfamiliar to patients, or patients may be unsure as to what particular PROM survey items are asking. Phone calls may help to address this potential issue and lead to an increase in compliance. This concept of personalized follow-up was further reinforced in one particular study by Tariq et al.,^12^ which used a last resort method of a personalized surgeon letter to individuals who did not initially respond to any interventions for follow up. They observed a 20% response rate in the intervention group as compared with 1.4% response rate in the control group that did not receive this letter.

We believe that this systematic review has strengths that may help to inform future orthopaedic research. We identified various patient-specific factors that may improve or reduce PROM compliance. In addition, this study was able to identify different means of intervention that could potentially lead to improved rates of compliance in PROMs collection.

It is important that orthopaedic researchers are aware of the potential impact that patient demographics may have on PROMs compliance. As reported within our study, male sex, extremes of age, and non-White race were cited as the most-common patient demographics associated with poor compliance rate. Early identification of these patients in the preoperative setting may be prudent, as focusing on these populations may generate different strategies that can be implemented to improve compliance within these groups moving forward. For example, in the younger population, it may be beneficial to obtain PROMs via SMS. As we move forward in a digital world in which the upcoming generations are being introduced to devices and internet access at a younger age, the use of electronic-based PROM surveys may soon become the norm.

Along these lines, orthopaedic researchers also should be aware of different interventions that may improve PROMs compliance. Patients can invariably be lost to follow-up for various reasons that may exist outside of a controlled research setting. As observed across many studies included in our review, phone calls, e-mails, and mail surveys represent successful methods that can lead to greater PROM response rates.

### Limitations

There are several limitations that should be considered. The initial review process was conducted with 2 independent reviewers with 2 rounds of the screening process. Although this study design allowed for greater discretion of the proposed inclusion criteria, it is still possible that several studies may have been excluded unknowingly. In addition, several studies that cleared the initial screening process were ultimately not included in the final analysis due to unclear descriptions of patient characteristics or response rates. The vast majority of studies included for analysis were observational cohort studies, either prospective or retrospective, thus demonstrating only Level II or III evidence. Only 5 of the 97 total studies were randomized controlled trials demonstrating Level I evidence. It is also important to note that while the scope of this review was broad across general orthopaedic research, this also led to a heterogeneity of study designs that made it difficult to assess differences between studies. Some studies used broad PROMs such as EQ-5D or Patient-Reported Outcomes Measurement Information System computer adaptive testing, whereas other studies reported subspecialty specific PROMs such as Boston Carpal Tunnel Questionnaire or the Oxford Hip and Knee Score. It is difficult to discern whether PROMs response rates may vary depending on the type of PROM that is used.

## Conclusions

The response rates for PROMs vary across the orthopaedic literature. Patient-specific factors, such as age (young or old) and race (non-White), may contribute to poor PROM response rate. Reminders and interventions significantly improve PROM response rates.

## Supplementary Data

ICMJE author disclosure forms

## References

[1] Gibbs D., Toop N., Grossbach A.J. (2023). Electronic versus paper patient-reported outcome measure compliance rates: A retrospective analysis. Clin Neurol Neurosurg.

[2] Basch E. (2017). Patient-Reported Outcomes—harnessing patients’ voices to improve clinical care. N Engl J Med.

[3] Squitieri L., Bozic K.J., Pusic A.L. (2017). The role of patient-reported outcome measures in value-based payment reform. Value Health.

[4] Maruszczyk K., Aiyegbusi O.L., Torlinska B., Collis P., Keeley T., Calvert M.J. (2022). Systematic review of guidance for the collection and use of patient-reported outcomes in real-world evidence generation to support regulation, reimbursement and health policy. J Patient Rep Outcomes.

[5] Porter M.E., Teisberg E.O. (2006).

[6] Siljander M.P., McQuivey K.S., Fahs A.M., Galasso L.A., Serdahely K.J., Karadsheh M.S. (2018). Current trends in patient-reported outcome measures in total joint arthroplasty: A study of 4 major orthopaedic journals. J Arthroplasty.

[7] Neve O.M., van Benthem P.P.G., Stiggelbout A.M., Hensen E.F. (2021). Response rate of patient reported outcomes: The delivery method matters. BMC Med Res Methodol.

[8] Gagnier J.J. (2017). Patient reported outcomes in orthopaedics. J Orthop Res.

[9] Foster A., Croot L., Brazier J., Harris J., O’Cathain A. (2018). The facilitators and barriers to implementing patient reported outcome measures in organisations delivering health related services: A systematic review of reviews. J Patient Rep Outcomes.

[10] Page M.J., McKenzie J.E., Bossuyt P.M. (2021). The PRISMA 2020 statement: An updated guideline for reporting systematic reviews. BMJ.

[11] Warwick H., Hutyra C., Politzer C. (2019). Small social incentives did not improve the survey response rate of patients who underwent orthopaedic surgery: A randomized trial. Clin Orthop Relat Res.

[12] Tariq M.B., Jones M.H., Strnad G., Sosic E., Spindler K.P., Cleveland Clinic OME Sports Health (2021). A last-ditch effort and personalized surgeon letter improves PROMs follow-up rate in sports medicine patients: A crossover randomized controlled trial. J Knee Surg.

[13] Yedulla N.R., Hester J.D., Patel M.M., Cross A.G., Peterson E.L., Makhni E.C. (2023). Pre-visit digital messaging improves patient-reported outcome measure participation prior to the orthopaedic ambulatory visit: Results from a double-blinded, prospective, randomized controlled trial. J Bone Joint Surg Am.

[14] Littlewood C., Bateman M., Butler-Walley S. (2021). Rehabilitation following rotator cuff repair: A multi-centre pilot & feasibility randomised controlled trial (RaCeR). Clin Rehabil.

[15] Schwartzenberger J., Presson A., Lyle A., O’Farrell A., Tyser A.R. (2017). Remote collection of patient-reported outcomes following outpatient hand surgery: A randomized trial of telephone, mail, and e-mail. J Hand Surg Am.

[16] Bot A.G.J., Anderson J.A., Neuhaus V., Ring D. (2013). Factors associated with survey response in hand surgery research. Clin Orthop Relat Res.

[17] Juto H., Gärtner Nilsson M., Möller M., Wennergren D., Morberg P. (2017). Evaluating non-responders of a survey in the Swedish fracture register: no indication of different functional result. BMC Musculoskelet Disord.

[18] Mellor X., Buczek M.J., Adams A.J., Lawrence J.T.R., Ganley T.J., Shah A.S. (2020). Collection of common knee patient-reported outcome instruments by automated mobile phone text messaging in pediatric sports medicine. J Pediatr Orthop.

[19] Randsborg P.H., Adamec D., Cepeda N.A., Pearle A., Ranawat A. (2021). Differences in baseline characteristics and outcome among responders, late responders, and never-responders after anterior cruciate ligament reconstruction. Am J Sports Med.

[20] Reinholdsson J., Kraus-Schmitz J., Forssblad M., Edman G., Byttner M., Stålman A. (2017). A non-response analysis of 2-year data in the Swedish Knee Ligament Register. Knee Surg Sports Traumatol Arthrosc.

[21] Zakaria H.M., Mansour T., Telemi E. (2019). Patient demographic and surgical factors that affect completion of patient-reported outcomes 90 days and 1 year after spine surgery: Analysis from the Michigan Spine Surgery Improvement Collaborative (MSSIC). World Neurosurg.

[22] Poulsen E., Lund B., Roos E.M. (2020). The Danish Hip Arthroscopy Registry: Registration completeness and patient characteristics between responders and non-responders. Clin Epidemiol.

[23] Borowsky P.A., Kadri O.M., Meldau J.E., Blanchett J., Makhni E.C. (2019). The remote completion rate of electronic patient-reported outcome forms before scheduled clinic visits—a proof-of-concept study using patient-reported outcome measurement information system computer adaptive test questionnaires. J Am Acad Orthop Surg Glob Res Rev.

[24] Zhang X.H., Li S.C., Xie F. (2012). An exploratory study of response shift in health-related quality of life and utility assessment among patients with osteoarthritis undergoing total knee replacement surgery in a tertiary hospital in Singapore. Value Health.

[25] Briggs M., Closs J.S. (1999). A descriptive study of the use of visual analogue scales and verbal rating scales for the assessment of postoperative pain in orthopedic patients. J Pain Symptom Manage.

[26] Jildeh T.R., Castle J.P., Abbas M.J., Dash M.E., Akioyamen N.O., Okoroha K.R. (2021). Age significantly affects response rate to outcomes questionnaires using mobile messaging software. Sports Med Arthrosc Rehabil Ther Technol.

[27] Paulsen A., Pedersen A.B., Overgaard S., Roos E.M. (2012). Feasibility of 4 patient-reported outcome measures in a registry setting. Acta Orthop.

[28] Schamber E.M., Takemoto S.K., Chenok K.E., Bozic K.J. (2013). Barriers to completion of patient reported outcome measures. J Arthroplasty.

[29] Lizzio V.A., Blanchett J., Borowsky P. (2019). Feasibility of PROMIS CAT administration in the ambulatory sports medicine clinic with respect to cost and patient compliance: A single-surgeon experience. Orthop J Sports Med.

[30] Robertsson O., Dunbar M.J. (2001). Patient satisfaction compared with general health and disease-specific questionnaires in knee arthroplasty patients. J Arthroplasty.

[31] Wagner M., Neururer S., Dammerer D. (2022). External validation of the Tyrolean hip arthroplasty registry. J Exp Orthop.

[32] Bernstein D.N., Karhade A.V., Bono C.M., Schwab J.H., Harris M.B., Tobert D.G. (2022). Sociodemographic factors are associated with patient-reported outcome measure completion in orthopaedic surgery: An analysis of completion rates and determinants among new patients. JB JS Open Access.

[33] Zhou X., Karia R., Iorio R., Zuckerman J., Slover J., Band P. (2014). Combined email and in-office technology improves patient reported outcomes collection in standard orthopaedic care. Osteoarthritis Cartilage.

[34] Sajak P.M., Aneizi A., Gopinath R. (2020). Factors associated with early postoperative survey completion in orthopaedic surgery patients. J Clin Orthop Trauma.

[35] Weir T.B., Zhang T., Jauregui J.J. (2021). Press Ganey surveys in patients undergoing upper-extremity surgical procedures: Response rate and evidence of nonresponse bias. J Bone Joint Surg Am.

[36] Kadiyala J., Zhang T., Aneizi A. (2022). Preoperative factors associated with 2-year postoperative survey completion in knee surgery patients. J Knee Surg.

[37] Kwon S.K., Kang Y.G., Chang C.B., Sung S.C., Kim T.K. (2010). Interpretations of the clinical outcomes of the nonresponders to mail surveys in patients after total knee arthroplasty. J Arthroplasty.

[38] Cotter E.J., Hannon C.P., Locker P. (2018). Male sex, decreased activity level, and higher BMI associated with lower completion of patient-reported outcome measures following ACL reconstruction. Orthop J Sports Med.

[39] Polk A., Rasmussen J.V., Brorson S., Olsen B.S. (2013). Reliability of patient-reported functional outcome in a joint replacement registry. A comparison of primary responders and non-responders in the Danish Shoulder Arthroplasty Registry. Acta Orthop.

[40] Nixon D.C., Zhang C., Weinberg M.W., Presson A.P., Nickisch F. (2020). Relationship of press ganey satisfaction and PROMIS function and pain in foot and ankle patients. Foot Ankle Int.

[41] Zhang T, Schneider MB, Weir TB (2023). Response bias for press ganey ambulatory surgery surveys after knee surgery. J Knee Surg.

[42] Roysam GS, Hill A, Jagonase L, Purushothaman B, Cross A, Lakshmanan P (2016). Two years following implementation of the British Spinal Registry (BSR) in a District General Hospital (DGH): perils, problems and PROMS. Spine J.

[43] Rothrock N., Barnard C., Bhatt S. (2018). Evaluation of the implementation of PROMIS CAT batteries for total joint arthroplasty in an electronic health record. Presentation at the International Society for Quality of Life Research. Dublin, Ireland, October 2018. Qual Life Res.

[44] Lovelock T., O’Brien M., Young I., Broughton N. (2018). Two and a half years on: data and experiences establishing a “Virtual Clinic” for joint replacement follow up. ANZ J Surg.

[45] Harris I.A., Peng Y., Cashman K. (2022). Association between patient factors and hospital completeness of a patient-reported outcome measures program in joint arthroplasty, a cohort study. J Patient Rep Outcomes.

[46] Bojcic J.L., Sue V.M., Huon T.S., Maletis G.B., Inacio M.C. (2014). Comparison of paper and electronic surveys for measuring patient-reported outcomes after anterior cruciate ligament reconstruction. Perm J.

[47] Besalduch-Balaguer M., Aguilera-Roig X., Urrútia-Cuchí G. (2015). Level of response to telematic questionnaires on Health Related Quality of Life on total knee replacement. Rev Esp Cir Ortop Traumatol.

[48] Bohm E.R., Kirby S., Trepman E. (2021). Collection and reporting of patient-reported outcome measures in arthroplasty registries: multinational survey and recommendations. Clin Orthop Relat Res.

[49] Triplet J.J., Momoh E., Kurowicki J., Villarroel L.D., Law T.Y., Levy J.C. (2017). E-mail reminders improve completion rates of patient-reported outcome measures. JSES Open Access.

[50] Molloy I.B., Yong T.M., Keswani A. (2020). Do medicare’s patient-reported outcome measures collection windows accurately reflect academic clinical practice. J Arthroplasty.

[51] Ling D.I., Finocchiaro A., Schneider B., Lai E., Dines J., Gulotta L. (2021). What factors are associated with patient-reported outcome measure questionnaire completion for an electronic shoulder arthroplasty registry. Clin Orthop Relat Res.

[52] Westenberg R.F., Nierich J., Lans J., Garg R., Eberlin K.R., Chen N.C. (2020). What factors are associated with response rates for long-term follow-up questionnaire studies in hand surgery. Clin Orthop Relat Res.

[53] Barnds B., Witt A., Orahovats A., Schlegel T., Hunt K. (2021). The effects of a pandemic on patient engagement in a patient-reported outcome platform at orthopaedic sports medicine centers (106). Orthop J Sports Med.

[54] Elsabeh R., Delgado K., Das K. (2021). Utilization of an automated SMS-based electronic patient-reported outcome tool in spinal surgery patients. Spine J.

[55] Perdomo-Pantoja A., Alomari S., Lubelski D. (2022). Implementation of an automated text message-based system for tracking patient-reported outcomes in spine surgery: an overview of the concept and our early experience. World Neurosurg.

[56] Barai A., Lambie B., Cosgrave C., Baxter J. (2018). Management of distal radius fractures in the emergency department: a long-term functional outcome measure study with the disabilities of arm, shoulder and hand (DASH) scores. Emerg Med Australas.

[57] Makhni E.C., Higgins J.D., Hamamoto J.T., Cole B.J., Romeo A.A., Verma N.N. (2017). Patient compliance with electronic patient reported outcomes following shoulder arthroscopy. Arthroscopy.

[58] Whitehouse M.R., Masri B.A., Duncan C.P., Garbuz D.S. (2015). Continued good results with modular trabecular metal augments for acetabular defects in hip arthroplasty at 7 to 11 years. Clin Orthop Relat Res.

[59] Clarnette R., Graves S., Lekkas C. (2016). Overview of the AOA national joint replacement registry. Orthop J Sports Med.

[60] Matthews A., Evans J.P. (2023). Evaluating the measures in patient-reported outcomes, values and experiences (EMPROVE study): a collaborative audit of PROMs practice in orthopaedic care in the United Kingdom. Ann R Coll Surg Engl.

[61] Franko O.I., London D.A., Kiefhaber T.R., Stern P.J. (2022). Automated reporting of patient outcomes in hand surgery: a pilot study. Hand (N Y).

[62] Shapiro L.M., Eppler S.L., Roe A.K., Morris A., Kamal R.N. (2021). The patient perspective on patient-reported outcome measures following elective hand surgery: a convergent mixed-methods analysis. J Hand Surg Am.

[63] Barker K.L., Frost H., MacDonald W.J., Fairbank J.C. (2010). Multidisciplinary rehabilitation or surgery for chronic low back pain - 7 year follow up of a randomised controlled trial: 25. Spine J Meeting Abstract.

[64] Bhatt S., Davis K., Manning D.W., Barnard C., Peabody T.D., Rothrock N.E. (2020). Integration of patient-reported outcomes in a total joint arthroplasty program at a high-volume academic medical center. J Am Acad Orthop Surg Glob Res Rev.

[65] Olach M. (2021). Feasibility of web-based patient-reported outcome assessment after arthroscopic knee surgery: the patients.

[66] Wylde V., Blom A.W., Whitehouse S.L., Taylor A.H., Pattison G.T., Bannister G.C. (2009). Patient-reported outcomes after total hip and knee arthroplasty: comparison of midterm results. J Arthroplasty.

[67] Tokish J.M., Chisholm J.N., Bottoni C.R., Groth A.T., Chen W., Orchowski J.R. (2017). Implementing an electronic patient-based orthopaedic outcomes system: factors affecting patient participation compliance. Mil Med.

[68] Rubery P. (2018). Standard of care PRO collection across a healthcare system. Qual Life Res.

[69] Thoma A., Levis C., Patel P., Murphy J., Duku E. (2018). Partial versus total trapeziectomy thumb arthroplasty: an expertise-based feasibility study. Plast Reconstr Surg Glob Open.

[70] Shu H.T., Bodendorfer B.M., Folgueras C.A., Argintar E.H. (2018). Follow-up compliance and outcomes of knee ligamentous reconstruction or repair patients enrolled in an electronic versus a traditional follow-up protocol. Orthopedics.

[71] Risberg M., Tryggestad C., Nordsletten L., Engebretsen L., Holm I. (2018). Active living with osteoarthritis implementation of evidence-based guidelines as first-line treatment for patients with knee and hip osteoarthritis. Osteoarthritis Cartil.

[72] Gakhar H., McConnell B., Apostolopoulos A.P., Lewis P. (2013). A pilot study investigating the use of at-home, web-based questionnaires compiling patient-reported outcome measures following total hip and knee replacement surgeries. J Long Term Eff Med Implants.

[73] van der Vliet Q.M.J., Sweet A.A.R., Bhashyam A.R. (2019). Polytrauma and high-energy injury mechanisms are associated with worse patient-reported outcomes after distal radius fractures. Clin Orthop Relat Res.

[74] Tilbury C., Leichtenberg C.S., Kaptein B.L. (2020). Feasibility of collecting multiple patient-reported outcome measures alongside the dutch arthroplasty register. J Patient Exp.

[75] Leonardsson O., Rolfson O., Hommel A., Garellick G., Åkesson K., Rogmark C. (2013). Patient-reported outcome after displaced femoral neck fracture: a national survey of 4467 patients. J Bone Joint Surg Am.

[76] Slover J.D., Karia R.J., Hauer C., Gelber Z., Band P.A., Graham J. (2015). Feasibility of integrating standardized patient-reported outcomes in orthopedic care. Am J Manag Care.

[77] Ho A., Purdie C., Tirosh O., Tran P. (2019). Improving the response rate of patient-reported outcome measures in an Australian tertiary metropolitan hospital. Patient Relat Outcome Meas.

[78] Ackerman I.N., Cavka B., Lippa J., Bucknill A. (2017). The feasibility of implementing the ICHOM standard set for hip and knee osteoarthritis: a mixed-methods evaluation in public and private hospital settings. J Patient Rep Outcomes.

[79] Pronk Y., Pilot P., Brinkman J.M., van Heerwaarden R.J., van der Weegen W. (2019). Response rate and costs for automated patient-reported outcomes collection alone compared to combined automated and manual collection. J Patient Rep Outcomes.

[80] Hajewski C., Anthony C.A., Rojas E.O., Westermann R., Willey M. (2019). Detailing postoperative pain and opioid utilization after periacetabular osteotomy with automated mobile messaging. J Hip Preserv Surg.

[81] Ross L.A., O’Rourke S.C., Toland G., MacDonald D.J., Clement N.D., Scott C.E.H. (2022). Loss to patient-reported outcome measure follow-up after hip arthroplasty and knee arthroplasty : patient satisfaction, associations with non-response, and maximizing returns. Bone Jt Open.

[82] Sepucha K.R., Atlas S.J., Chang Y. (2018). Informed, patient-centered decisions associated with better health outcomes in orthopedics: prospective cohort study. Med Decis Making.

[83] Spindler K., Jin Y., Jones M. (2022). Paper 86: Symptoms of post-traumatic osteoarthritis remain stable up to 10 years after ACL reconstruction. Orthopaedic J Sports Med.

[84] Fitzpatrick R., Morris R., Hajat S. (2000). The value of short and simple measures to assess outcomes for patients of total hip replacement surgery. Qual Health Care.

[85] Tariq M.B., Vega J.F., Westermann R., Jones M., Spindler K.P. (2019). Arthroplasty studies with greater than 1000 participants: analysis of follow-up methods. Arthroplast Today.

[86] Bell K., Warnick E., Nicholson K. (2018). Patient adoption and utilization of a web-based and mobile-based portal for collecting outcomes after elective orthopedic surgery. Am J Med Qual.

[87] Palmen L.N., Schrier J.C., Scholten R., Jansen J.H., Koëter S. (2016). Is it too early to move to full electronic PROM data collection?: A randomized controlled trial comparing PROM’s after hallux valgus captured by e-mail, traditional mail and telephone. Foot Ankle Surg.

[88] Fife J., McGee A., Swantek A., Makhni E. (2022). Paper 78: Integrating PROM Collection for Shoulder Surgical Patients through the Electronic Medical Record: A Low Cost and Effective Strategy for High Fidelity PROM Collection. Orthopaed J Sports Med.

[89] Harris K.K., Dawson J., Jones L.D., Beard D.J., Price A.J. (2013). Extending the use of PROMs in the NHS—using the Oxford Knee Score in patients undergoing non-operative management for knee osteoarthritis: a validation study. BMJ Open.

[90] Haskell A., Kim T. (2018). Implementation of patient-reported outcomes measurement information system data collection in a private orthopedic surgery practice. Foot Ankle Int.

[91] Whitehouse S.L., Blom A.W., Taylor A.H., Pattison G.T., Bannister G.C. (2005). The Oxford Knee Score; problems and pitfalls. Knee.

[92] Papakostidou I., Dailiana Z.H., Papapolychroniou T. (2012). Factors affecting the quality of life after total knee arthroplasties: a prospective study. BMC Musculoskelet Disord.

[93] Marx R.G., Wolfe I.A., Turner B.E., Huston L.J., Taber C.E., Spindler K.P. (2022). MOON’s strategy for obtaining over eighty percent follow-up at 10 years following ACL reconstruction. J Bone Joint Surg Am.

[94] Baeesa S.S. (2015). Cervical disc arthroplasty for degenerative disc disease: two-year follow-up from an international prospective, multicenter, observational study. Spine J.

[95] Rolfson O., Salomonsson R., Dahlberg L.E., Garellick G. (2011). Internet-based follow-up questionnaire for measuring patient-reported outcome after total hip replacement surgery-reliability and response rate. Value Health.

[96] Owen R.J., Zebala L.P., Peters C., McAnany S. (2018). PROMIS Physical function correlation with NDI and mJOA in the surgical cervical myelopathy patient population. Spine (Phila Pa 1976).

[97] Pronk Y., van der Weegen W., Vos R., Brinkman J.M., van Heerwaarden R.J., Pilot P. (2020). What is the minimum response rate on patient-reported outcome measures needed to adequately evaluate total hip arthroplasties. Health Qual Life Outcomes.

[98] Scott E.J., Anthony C.A., Rooney P., Lynch T.S., Willey M.C., Westermann R.W. (2020). Mobile phone administration of hip-specific patient-reported outcome instruments correlates highly with in-office administration. J Am Acad Orthop Surg.

[99] Nguyen J., Marx R., Hidaka C., Wilson S., Lyman S. (2017). Validation of electronic administration of knee surveys among ACL-injured patients. Knee Surg Sports Traumatol Arthrosc.

[100] Owen R.J., Khan A.Z., McAnany S.J., Peters C., Zebala L.P. (2019). PROMIS correlation with NDI and VAS measurements of physical function and pain in surgical patients with cervical disc herniations and radiculopathy. J Neurosurg Spine.

[101] Sierakowski K.L., Dean N.R., Mohan R., John M., Griffin P.A., Bain G.I. (2020). Prospective randomized cohort study to explore the acceptability of patient-reported outcome measures to patients of hand clinics. J Hand Surg Glob Online.

[102] Nagappa M., Querney J., Martin J. (2021). Perioperative satisfaction and health economic questionnaires in patients undergoing an elective hip and knee arthroplasty: a prospective observational cohort study. Anesth Essays Res.

[103] Karia R., Slover J., Hauer C., Gelber Z., Band P.., Graham J. (2013). Networking to capture patient-reported outcomes during routine orthopaedic care across two distinct institutions. Osteoarthritis Cartil.

[104] Slover J.D., Karia R.J., Hauer C., Gelber Z., Band P.A., Graham J. (2015). Feasibility of integrating standardized patient-reported outcomes in orthopedic care. Am J Manag Care.

[105] Lyman S., Hidaka C., Fields K., Islam W., Mayman D. (2020). Monitoring patient recovery after THA or TKA using mobile technology. HSS J.

[106] Olley L.M., Carr A.J. (2008). The use of a patient-based questionnaire (the Oxford Shoulder Score) to assess outcome after rotator cuff repair. Ann R Coll Surg Engl.

[107] Shapiro L.M., Đình M.P., Tran L., Fox P.M., Richard M.J., Kamal R.N. (2022). Short message service-based collection of patient-reported outcome measures on hand surgery global outreach trips: a pilot feasibility study. J Hand Surg Am.

